# The feasibility of web surveys for obtaining patient-reported outcomes from cancer survivors: a randomized experiment comparing survey modes and brochure enclosures

**DOI:** 10.1186/s12874-019-0859-9

**Published:** 2019-11-15

**Authors:** Morgan M. Millar, Joanne W. Elena, Lisa Gallicchio, Sandra L. Edwards, Marjorie E. Carter, Kimberly A. Herget, Carol Sweeney

**Affiliations:** 1 Department of Internal Medicine, 295 Chipeta Way University of Utah, Salt Lake City, UT 84132 USA; 2 Utah Cancer Registry, University of Utah, 250 E 200 S, Suite 1375, Salt Lake City, UT 84111 USA; 3 Epidemiology and Genomics Research Program, Division of Cancer Control and Population Sciences, National Cancer Institute, National Institutes of Health, 9609 Medical Center Drive, Rockville, MD 20852 USA; 4 Huntsman Cancer Institute, University of Utah, 2000 Cir of Hope Dr. Salt, Lake City, UT 84112 USA

**Keywords:** Response rate, Surveys, Survey mode, Web surveys, Web-push design, Patient-reported outcomes, Cancer survivors, Cancer registry

## Abstract

**Background:**

Central cancer registries are often used to survey population-based samples of cancer survivors. These surveys are typically administered via paper or telephone. In most populations, web surveys obtain much lower response rates than paper surveys. This study assessed the feasibility of web surveys for collecting patient-reported outcomes via a central cancer registry.

**Methods:**

Potential participants were sampled from Utah Cancer Registry records. Sample members were randomly assigned to receive a web or paper survey, and then randomized to either receive or not receive an informative brochure describing the cancer registry. We calculated adjusted risk ratios with 95% confidence intervals to compare response likelihood and the demographic profile of respondents across study arms.

**Results:**

The web survey response rate (43.2%) was lower than the paper survey (50.4%), but this difference was not statistically significant (adjusted risk ratio = 0.88, 95% confidence interval = 0.72, 1.07). The brochure also did not significantly influence the proportion responding (adjusted risk ratio = 1.03, 95% confidence interval = 0.85, 1.25). There were few differences in the demographic profiles of respondents across the survey modes. Older age increased likelihood of response to a paper questionnaire but not a web questionnaire.

**Conclusions:**

Web surveys of cancer survivors are feasible without significantly influencing response rates, but providing a paper response option may be advisable particularly when surveying older individuals. Further examination of the varying effects of brochure enclosures across different survey modes is warranted.

## Background

Central cancer registries, which are mandated to collect data on all reportable cancer diagnoses within a defined geographic area for public health surveillance purposes [1–3], are an important resource for researchers interested in ascertaining and recruiting individuals who have been diagnosed with cancer [4–11]. Central cancer registries in U.S. states are population-based—that is, they are required to meet standards of complete ascertainment of incident cancers [12, 13]—and thus provide unbiased sample frames of cancer survivors in their catchment areas [14, 15]. These registries have been utilized for a variety of studies, including obtaining patient-reported outcomes and assessing quality-of-life among cancer survivors [16, 17], investigating etiology [18], identifying outcomes of treatment [19], and promoting preventive behaviors [20].

There is some concern about the potential for low participation rates when surveying through cancer registries [21, 22]. Generally speaking, survey response rates have been declining over time [23–28], and there is some evidence that lower response will result in more nonresponse bias [29]. Numerous studies have documented concerns about demographic differences between those who do not respond and those who do participate [22, 26–31]. In a recent evaluation of 10 years of recruitment efforts conducted via a central cancer registry, we found that a number of study-related and individual demographic variables predicted response outcomes [32]. Some studies have utilized randomized designs to evaluate the effect of survey administration methods used by registries on response, including sending the questionnaire in the initial recruitment packet rather than first obtaining consent [33], questionnaire length [7], type and amount of incentives offered [6, 7], and inclusion of phone contacts in addition to letters [9].

Generally speaking, web surveys have become popular for the advantages they offer in terms of lower costs, quicker data collection, automatic data entry, and the ability to require responses to all questions. However, across a variety of populations, response rates to web surveys have long been lower than those to paper surveys [34–39]. The feasibility of web-based surveys in registry-based research has not been evaluated, which may in part be due to the fact that registries do not routinely collect email addresses of cancer patients. However, survey researchers have increasingly adopted alternative strategies for administering web surveys when email addresses are not available. One such strategy that has been used in a variety of populations is the web-push design, which uses postal mail to contact sample members and encourage response to a web questionnaire, while withholding a paper response option until later in the survey cycle [40]. Given recent research showing the success of this approach, as well as the potential web surveys have for increased data quality and quicker data processing, we sought to assess the feasibility of a postal-mail administered web survey to collect patient-reported outcomes via a central cancer registry. Using a randomized design, we compared response to a web survey to that of a paper survey in a population of individuals diagnosed with cancer ascertained via a cancer registry and examined the demographic profile of respondents for each mode.

As a secondary research question, we also assessed the effect of including an informative brochure describing the cancer registry on response outcomes. Such brochures, which explain how a person’s name was obtained for the study, are required by some registries when contacting cancer survivors for research recruitment. Our registry has not previously utilized such a brochure, so we aimed to examine its effects on response in order to inform future procedures and provide guidance to other registries for maximizing recruitment outcomes.

## Methods

### Sample

The population of interest for this study was Utah residents diagnosed as adults (age 20 or older) from 2001 to 2016 with colorectal, breast (female only), prostate, ovarian, and multiple myeloma cancers, as reported to the Utah Cancer Registry. We excluded in situ colorectal cancer from our eligibility criteria, because these individuals may not be aware of their cancer diagnosis. Otherwise, cancers of all stages were included. Cancer stage was defined using Surveillance, Epidemiology, and End Results Program (SEER) summary stage 2000 or derived SEER summary stage 2000 [41].

Eligible individuals were those who were considered early-age-onset, defined as under 50 at time of diagnosis for breast or colorectal cancer, under 55 for prostate, and under 65 for multiple myeloma or ovarian cancer. This study focused on early-onset cancer diagnoses in part because these individuals are likely to be survivors for a relatively long time compared to those diagnosed at older ages, and also because of a growing recognition of the long-term, unique experiences of cancer survivorship among those diagnosed at early ages [42]. These include financial hardship, psychological distress, and other health complications [43–48].

The study also considered two groups of cancer survivors, defined by time since diagnosis. The first group included those recently diagnosed and the second were longer-term survivors. Recently diagnosed was defined as cases reported to the cancer registry within the 12 months preceding the study start date (September, 2016). Longer-term survivor equated to greater than 5 years post-diagnosis for those with colorectal, breast, and prostate cancer, and greater than 3 years for ovarian cancer and multiple myeloma. We used stratified random sampling (stratified by time since diagnosis and cancer site) to select cases for the study.

All races/ethnicities and individuals from all parts of Utah were included. We oversampled Hispanics and residents of rural counties (using the Rural-Urban Continuum Codes [49], we coded each county as metropolitan or non-metropolitan [rural]) among the long-term survivors, doubling the proportion selected for these groups compared to their representation in the Utah population. We did not use ethnicity and rurality as part of the sampling design among the recently diagnosed patients because demographic information for these individuals was incomplete at the time of study selection. The initial sample included 470 individuals. The distribution of these sampled individuals across 10 strata as follows: long-term colorectal: 63, long-term myeloma: 33, long-term breast: 33, long-term ovarian: 33, long-term prostate: 38, recently diagnosed colorectal: 68, recently diagnosed myeloma: 33, recently diagnosed breast: 69, recently diagnosed ovarian: 33, recently diagnosed prostate: 67.

### Experimental design

**Fig. 1:**
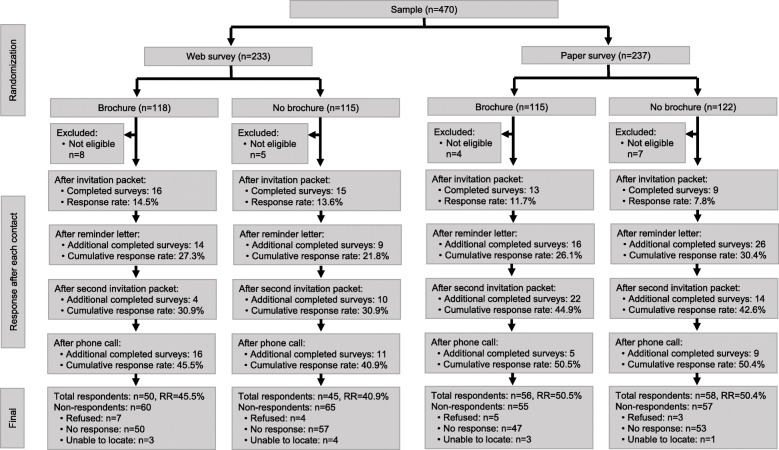
Experimental randomization and response outcomes for a survey of Utah cancer survivors^1.^ 1 Because the pre-notification letter did not yet elicit responses, it is not included in the figure

After selection based on the stratified design noted above, all sampled individuals were pooled and then randomly assigned 1:1 to one of two experimental arms to compare outcomes by survey mode: to receive either a paper or web questionnaire. Within each survey mode experimental arm, individuals were then randomly assigned 1:1 to either receive a brochure or not receive a brochure in the first mailing. Figure 1 displays the experimental design, sample randomization, recruitment outcomes after each contact, and final case dispositions for the study.

### Materials

The questionnaire was newly developed for this study. It consisted of up to 35 items including questions on current health, cancer recurrence, and willingness to participate in various kinds of cancer research (see Additional file 1). The web-based instrument was constructed using Qualtrics survey software [50]. Following a unified mode design approach, we formatted a paper questionnaire to visually resemble the web-based instrument as much as possible to reduce mode effects [51]. On the paper questionnaire, each item was enclosed in a box to resemble the page-by-page display of the web instrument. The same imagery was used on the paper questionnaire cover and the welcome screen of the web instrument.

A brochure describing the role of a central cancer registry and its involvement with research activities was also designed to inform individuals about the entity contacting them and how their name was selected for inclusion in a study. This brochure was unique from most brochures used in recruitment in that it was not specific to the study being conducted, but contained more general information about the cancer registry. The brochure incorporated similar imagery from the questionnaire.

### Survey administration

We utilized a series of multiple contacts to request survey participation. For both survey modes, the initial mode of contact was postal mail, as email addresses are not routinely obtained in cancer registry reports. Potential participants received up to four mailings (pre-notification letter with or without brochure, invitation packet with either questionnaire and stamped return envelope or web survey instructions, thank-you/reminder letter, and a replacement packet). All mailings utilized official University of Utah letterhead and envelopes, as well as postage stamps for outgoing and return envelope postage. For the paper survey arm, each of these contacts requested response by paper questionnaire, and the web response option was not offered. For the web arm, all of these contacts only mentioned response via the web-based questionnaire, and unlike the standard web-push approach, a paper response option was never offered. Telephone calls were made to nonresponding individuals as the last stage of the recruitment protocol. Up to three call attempts were made at varying times of day and days of the week to reach each nonresponder. In these calls, nonresponding individuals in both study arms were encouraged to respond via the mode they were assigned, and also offered the option of responding via telephone.

Individuals identified as Hispanic in the registry database were sent bilingual English and Spanish invitation letters. For the paper survey arm, the mailed questionnaires were in English; the accompanying letter noted that a Spanish version could be sent upon request. Web respondents could select to respond via a Spanish version of the questionnaire on the survey home screen.

To simplify the online response process for those assigned to the web survey, we utilized a URL shortener to create a simplified, meaningful survey web address for respondents to be able to easily type into their web browsers from the paper letters they received in the mail. Each sample member assigned to the web survey also received a 6-digit numerical access code for logging into the survey. The URL and individualized access code were provided in each mailing to the web survey arm except for the pre-notification letter.

### Statistical analysis

Counts and percentages for demographic and cancer variables were calculated for the full eligible sample and separately by assigned survey mode. Response rates (proportion responding) were calculated for the full sample as well as for demographic/cancer subgroups using the number of sample members that returned completed questionnaires divided by the sample size minus ineligible individuals, in accordance with Response Rate 1 guidelines outlined by the American Association for Public Opinion Research [52]. Web survey breakoffs were not counted as responses. Chi-square tests were used to test for differences in sample allocation and response by each demographic and cancer variable. Adjusted risk ratios (RR) with 95% confidence intervals (CI) were calculated to determine the relationship between each treatment (web compared to paper survey mode and brochure delivery compared to no brochure) and each demographic or cancer variable and the binary outcome of survey response (responded compared to did not respond) while accounting for demographic and cancer variables. Risk ratios were obtained using Poisson regression with a robust variance estimate [53, 54]. All calculations were performed using Stata MP Version 13.1 [55].

## Results

Twenty-four individuals were determined to be ineligible for the study after randomization and were not included in any further analysis. Reasons for ineligibility included: individuals we later learned had been deceased at the time of study selection, cases sampled shortly after diagnosis that were later determined (after the registry’s standard case coding and editing process) to not have been diagnosed with a reportable cancer, and individuals ineligible for study inclusion according to registry policy for contacting cases (e.g., individuals who had previously requested that the registry not contact them regarding participation in research studies). This resulted in a working sample of 446 cancer survivors.

Table 1 describes the eligible sample overall and by survey mode. Among all eligible sampled individuals, 56.5% were female, a majority were between ages 40 and 59 (66.6%), 6.7% were nonwhite, 15.5% were Hispanic, and 13.7% resided in rural counties. By cancer site, the eligible sample was 28.7% colorectal cancer, 14.4% multiple myeloma, 21.8% breast cancer, 21.1% prostate cancer, and 14.1% ovarian cancer. Local stage at diagnosis was most common (47.3%), followed by regional (25.6%), distant (21.8%), and in situ (5.4%). The samples within each of the experimental arms did not differ significantly in terms of any demographic (sex, age, race, ethnicity, geography) or cancer (time since diagnosis, cancer site, or stage at diagnosis) variables.

**Table 1:** Characteristics of the eligible sample for a survey of Utah cancer survivors

	Full sample		Paper		Web		*P*
	N	Col. %	N	Col. %	N	Col. %	
All cases	446	100.0	226	100.0	220	100.0	
Time since diagnosis^1^							0.805
Long-term	192	43.0	96	42.5	96	43.6	
Short-term	254	57.0	130	57.5	124	56.4	
Sex							0.803
Female	252	56.5	129	57.1	123	55.9	
Male	194	43.5	97	42.9	97	44.1	
Current age							0.600
< 40	74	16.6	35	15.5	39	17.7	
40–49	126	28.3	68	30.1	58	26.4	
50–59	171	38.3	82	36.3	89	40.5	
> =60	75	16.8	41	18.1	34	15.5	
Diagnosis age							0.882
< 40	102	22.9	53	23.5	49	22.3	
40–49	212	47.5	104	46.0	108	49.1	
50–59	98	22.0	50	22.1	48	21.8	
> =60	34	7.6	19	8.4	15	6.8	
Race^2^							0.650
White	416	93.3	212	93.8	204	92.7	
Nonwhite	30	6.7	14	6.2	16	7.3	
Ethnicity^3^							0.903
Hispanic	68	15.5	34	15.3	34	15.8	
Non-Hispanic	372	84.5	189	84.8	183	84.3	
Geography							0.093
Rural	61	13.7	37	16.4	24	10.9	
Metropolitan	385	86.3	189	83.6	196	89.1	
Cancer site							0.992
Colorectal	128	28.7	63	27.9	65	29.6	
Myeloma	64	14.4	32	14.2	32	14.6	
Breast	97	21.8	51	22.6	46	20.9	
Ovarian	63	14.1	32	14.2	31	14.1	
Prostate	94	21.1	48	21.2	46	20.9	
Stage at diagnosis							0.707
In situ	24	5.4	10	4.4	14	6.4	
Localized	211	47.3	111	49.1	100	45.5	
Regional	114	25.6	55	24.3	59	26.8	
Distant^4^	97	21.8	50	22.1	47	21.4	

1. “Long-term” survivors were defined as > 5 years from diagnosis for breast, colorectal, and prostate and > 3 years for myeloma and ovarian cancer. “Short-term” survivors were those < 1 year from diagnosis as of sample selection (September 2016)

2. Due to small counts, all races other than white have been grouped for analysis

3. Ethnicity could not be established for 6 individuals. Percentages based on total number of survivors with ethnicity data

4. Because fewer than 5 sample members were unstaged, they have been grouped together with distant stage to maintain confidentiality

Two-hundred and nine of the 446 eligible individuals completed the survey, resulting in a response rate of 46.9%. Eleven individuals (2.5%) could not be contacted due to outdated contact information, 19 (4.3%) refused participation, and the remaining 207 (46.4%) did not respond. Figure 1 displays response outcomes at each stage of the contact protocol by experimental arm. Across all study arms combined, each subsequent contact yielded additional responses: 25.4% of responses were obtained after the first invitation packet, 31.1% after the reminder letter, 23.9% after the second/replacement invitation packet, and 19.6% after the final, phone call stage.

Table 2 displays response rates by experimental treatment, along with adjusted risk ratios and 95% confidence intervals for the outcome of survey response by each experimental treatment. For the comparison of survey mode, we found that the web survey response rate was 43.2%, compared to 50.4% for paper, but this difference was not statistically significant (RR = 0.88, 95% CI = 0.72, 1.07). The use of a brochure in the recruitment materials also did not significantly influence the proportion responding; 48.0% of those assigned to the brochure treatment responded compared to 45.8% of those not sent a brochure (RR = 1.03, 95% CI = 0.85, 1.25). Although not significant, the brochure appeared to have a more positive effect among the web arm (response rate was almost 5 percentage points higher) than the paper arm, in which response was unchanged.

**Table 2:** Response outcomes by survey mode and brochure enclosure, survey of Utah cancer survivors

	Sample	Responded^1^		Adjusted Risk Ratio^2^	95% CI
	*n*	*n*	%		
Overall	446	209	46.9		
Survey mode					
Paper	226	114	50.4	1.00	Ref.
Web	220	95	43.2	0.88	0.72, 1.07
Brochure					
No	225	103	45.8	1.00	Ref.
Yes	221	106	48.0	1.03	0.85, 1.25
Brochure by mode^3^					
Paper, no brochure	115	58	50.4	1.00	Ref.
Paper, brochure	111	56	50.5	0.97	0.75, 1.25
Web, no brochure	110	45	40.9	1.00	Ref.
Web, brochure	110	50	45.5	1.08	0.81, 1.45

1. The percentage of cases that responded equates to response rate (AAPOR RR1)

2. Risk ratios from multivariable models predicting survey response that include design features and adjust for time since diagnosis, sex, age, race, ethnicity, geography, cancer site, and cancer stage at diagnosis. In these models, none of the demographic or cancer variables significantly predicted response

3. When conducting the model using a single reference group to compare all four mode-brochure combinations simultaneously, the adjusted risk ratios were as follows: Paper, no brochure: Ref.; Paper, brochure: RR = 0.98 (95% CI: 0.76, 1.27); Web, no brochure: RR = 0.83 (95% CI: 0.62, 1.10); Web, brochure: RR = 0.91 (95% CI: 0.70, 1.18)

Bivariate comparisons of the demographics of respondents to non-respondents for the full sample as well as by mode are displayed in Table 3. For the full sample, there was some variation between respondents and nonrespondents for several demographic variables. Individuals diagnosed within the year prior to selection had a higher response rate than those considered long-term survivors (51.6% vs. 40.6%, *P* = 0.022). Individuals aged under 40 were underrepresented among respondents (36.5% response rate) compared to older individuals; those aged 60 or older had the highest response rate of all age categories (58.7%; *P* = 0.028). The response rate was also significantly higher for non-Hispanics than Hispanics (49.2% compared to 33.8%, *P* = 0.020). There were no significant differences between respondents and nonrespondents in terms of sex, race, rurality, cancer site, or stage at diagnosis.

**Table 3:** Comparison of respondents to non-respondents in a survey of Utah cancer survivors^1,2^

	Full sample					Paper					Web				
	Non-respondents		Respondents			Non-respondents		Respondents			Non-respondents		Respondents		
	n	%	n	%	*P*	n	%	n	%	*P*	n	%	n	%	*P*
Time since diagnosis					**0.022**					0.144					0.076
Long-term	114	59.4	78	40.6		53	55.2	43	44.8		61	63.5	35	36.5	
Recent	123	48.4	131	51.6		59	45.4	71	54.6		64	51.6	60	48.4	
Sex					0.130					**0.016**					0.760
Female	126	50.0	126	50.0		55	42.6	74	57.4		71	57.7	52	42.3	
Male	111	57.2	83	42.8		57	58.8	40	41.2		54	55.7	43	44.3	
Current age					**0.028**					0.223					0.139
< 40	47	63.5	27	36.5		21	60.0	14	40.0		26	66.7	13	33.3	
40–49	62	49.2	64	50.8		34	50.0	34	50.0		28	48.3	30	51.7	
50–59	97	56.7	74	43.3		42	51.2	40	48.8		55	61.8	34	38.2	
> =60	31	41.3	44	58.7		15	36.6	26	63.4		16	47.1	18	52.9	
Race					0.055					0.558					**0.040**
White	216	51.9	200	48.1		104	49.1	108	50.9		112	54.9	92	45.1	
Nonwhite	21	70.0	9	30.0		8	57.1	6	42.9		13	81.3	< 5^3^	–	
Ethnicity					**0.020**					0.439					**0.011**
Hispanic	45	66.2	23	33.8		19	55.9	15	44.1		26	76.5	8	23.5	
Non-Hispanic	189	50.8	183	49.2		92	48.7	97	51.3		97	53.0	86	47.0	
Geography					0.909					0.904					0.874
Rural	32	52.5	29	47.5		18	48.7	19	51.4		14	58.3	10	41.7	
Metro-politan	205	53.3	180	46.8		94	49.7	95	50.3		111	56.6	85	43.4	
Cancer site					0.144					0.074					0.728
Colorectal	77	60.2	51	39.8		37	58.7	26	41.3		40	61.5	25	38.5	
Myeloma	31	48.4	33	51.6		12	37.5	20	62.5		19	59.4	13	40.6	
Breast	51	52.6	46	47.4		24	47.1	27	52.9		27	58.7	19	41.3	
Ovarian	26	41.3	37	58.7		11	34.4	21	65.6		15	48.4	16	51.6	
Prostate	52	55.3	42	44.7		28	58.3	20	41.7		24	52.2	22	47.8	
Stage					0.174					0.162					0.652
In situ	10	41.7	14	58.3		< 5^3^	–	7	70.0		7	50.0	7	50.0	
Localized	118	55.9	93	44.1		57	51.4	54	48.7		61	61.0	39	39.0	
Regional	65	57.0	49	43.0		32	58.2	23	41.8		33	55.9	26	44.1	
Distant	44	45.4	53	54.6		20	40.0	30	60.0		24	51.1	23	48.9	

1. Percent of respondents equates to response rate

2. *P*-values for statistically significant comparisons are presented in boldface.

3. Cell counts less than five are masked for protection of confidentiality

Among the paper arm, respondents and nonrespondents differed significantly only in terms of sex; 57.4% of females in the paper arm responded compared to only 41.2% of men (*P* = 0.016). However, this same trend was not observed for the web group, in which men and women responded at similar levels (42.3 and 44.3%, respectively). When comparing these response rates for females across modes, we found the response rate for females assigned to the paper survey arm was significantly higher than the response among females assigned to the web (*P* = 0.017). In the web arm, two variables showed significant differences between responders and nonresponders: race and ethnicity. Nonwhites and Hispanics were both underrepresented among respondents (*P* = 0.040 and *P* = 0.011 respectively).

Our multivariable assessment of the demographic representativeness of the responding samples and demographic-specific response rates by survey mode (Table 4) found few differences between modes. Adjusted risk ratios for response among the paper arm show the only significant predictors to be age 60 or above, with increased likelihood of response compared to age under 40 (RR: 1.78, 95% CI: 1.02, 3.06) and time since diagnosis (recently diagnosed RR: 1.36, 95% CI: 1.01, 1.84). The only variable significantly associated with response among the web arm in the adjusted model was Hispanic ethnicity (RR: 0.51, 95% CI: 0.27, 0.96). While we were unable to assess response outcomes by educational attainment because this information is not available in the registry database, we did collect educational attainment information in the questionnaire. The distribution of respondents by level of education was similar across study arms; 46.0% of paper respondents and 46.3% of web respondents reported having a bachelor’s degree or higher, 34.5% of paper and 28.4% of web respondents reported some college/associate’s degree, and 19.5% of paper and 25.3% of web respondents reported having a high school degree or less (*P* = 0.500).

**Table 4:** Demographic representativeness of respondents by survey mode, survey of Utah cancer survivors^1^

	Full sample		Paper					Web				
			Respondents		Adj. RR^2^	95% CI	*P*	Respondents		Adj. RR^2^	95% CI	*P*
	n	Col.%	n	Col.%				n	Col.%			
Time since diagnosis												
Long-term	192	43.0	43	37.7	1.00	Ref.		35	36.8	1.00	Ref.	
Short-term	254	57.0	71	62.3	1.36	1.01, 1.84	**0.043**	60	63.2	1.07	0.74, 1.54	0.719
Sex												
Female	252	56.5	74	64.9	1.39	0.91, 2.12	0.127	52	54.7	0.75	0.46, 1.22	0.249
Male	194	43.5	40	35.1	1.00	Ref.		43	45.3	1.00	Ref.	
Current age												
< 40	74	16.6	14	12.3	1.00	Ref.		13	13.7	1.00	Ref.	
40–49	126	28.3	34	29.8	1.34	0.83, 2.16	0.238	30	31.6	1.49	0.86, 2.59	0.157
50–59	171	38.3	40	35.1	1.67	0.99, 2.80	0.053	34	35.8	1.06	0.59, 1.89	0.856
> =60	75	16.8	26	22.8	1.78	1.02, 3.06	**0.043**	18	19.0	1.56	0.80, 3.06	0.196
Race												
White	416	93.3	108	94.7	1.00	Ref.		92	96.8	1.00	Ref.	
Nonwhite	30	6.7	6	5.3	0.76	0.41, 1.41	0.379	< 5^3^	–	0.45	0.16, 1.29	0.138
Ethnicity												
Hispanic	68	15.5	15	13.4	0.90	0.60, 1.35	0.611	8	8.5	0.51	0.27, 0.96	**0.036**
Non-Hispanic	372	84.6	97	86.6	1.00	Ref.		86	91.5	1.00	Ref.	
Geography												
Rural	61	13.7	19	16.7	0.84	0.57, 1.21	0.347	10	10.5	0.91	0.55, 1.51	0.728
Metropolitan	385	86.3	95	83.3	1.00	Ref.		85	89.5	1.00	Ref.	
Cancer site												
Colorectal	128	28.7	26	22.8	1.00	Ref.		25	26.3	1.00	Ref.	
Myeloma	64	14.4	20	17.5	1.45	0.77, 2.73	0.256	13	13.7	0.78	0.40, 1.54	0.480
Breast	97	21.8	27	23.7	0.92	0.57, 1.50	0.745	19	20.0	1.18	0.63, 2.21	0.597
Ovarian	63	14.1	21	18.4	1.26	0.79, 2.03	0.334	16	16.8	1.51	0.85, 2.68	0.157
Prostate	94	21.1	20	17.5	0.89	0.51, 1.56	0.687	22	23.2	1.22	0.72, 2.04	0.459
Stage												
In situ	24	5.4	7	6.1	1.00	Ref.		7	7.4	1.00	Ref.	
Localized	211	47.3	54	47.4	0.76	0.44, 1.30	0.313	39	41.1	0.74	0.37, 1.46	0.383
Regional	114	25.6	23	20.2	0.60	0.34, 1.07	0.086	26	27.4	0.86	0.43, 1.71	0.667
Distant	97	21.8	30	26.3	0.59	0.28, 1.21	0.149	23	24.2	1.11	0.52, 2.39	0.786

1. *P*-values for statistically significant comparisons are presented in boldface

2. Adjusted (Adj.) risk ratios from a multivariable model that adjusts for other variables in table

3. Cell counts less than five are masked for protection of confidentiality

## Discussion

In this randomized comparison of web and paper survey response outcomes in a study of cancer survivors ascertained through a central cancer registry, the overall proportion responding was slightly lower among the web arm. However, this difference in response rates was not significant. Considering that a recently-updated meta-analysis demonstrated that web surveys continue to yield response rates that are 12 percentage-points lower than other modes [39], the difference in response rates in this study (a seven percentage-point difference) was smaller than anticipated. This is a notable departure from longstanding trends showing web surveys obtaining much lower response rates than paper-based surveys [34–38, 56] except in limited instances with specialized populations wherein Internet use may be more prevalent, including college students [57], physicians [58] [59], and volunteer samples recruited online [60].

Furthermore, unlike most prior research showing the demographic profile of web survey respondents is often much different and less representative of the target population than is found with paper surveys [61–66], in this study the demographic representativeness of the responding sample members was mostly similar across survey modes. However, we did find that the oldest age group (65 or above) was more likely to respond than the youngest among the paper arm, but we did not observe this for web. This is consistent with prior research finding older individuals overrepresented among responders to a paper survey while web respondents are on average younger [63, 65] and that responders to web surveys are typically younger than those to a paper survey [64]. Due to the continuing relationship between age and web survey response patterns, using web alone to survey cancer survivors may not yet be advisable, especially since the larger population of cancer survivors is on average older than those included in this study, and only 44% of adults age 80 or above use the internet [67]. We also observed that Hispanics were less likely than non-Hispanics to respond the web survey, but this was not the case for the paper survey. This further suggests that adding a paper response option would be beneficial.

Our web survey was slightly different than most in that it was not email-administered. The use of a primarily postal mail-based contact protocol to encourage response may have been advantageous in helping to establish legitimacy and trust in the surveyor [40], but we did not test this directly. In the final phone call follow-up phase of this study, we offered nonrespondents in either study arm the option to respond over the telephone, but only 3 individuals completed the survey using this method. A similar approach that uses a paper response option for nonresponders to a web survey, the web-push design, has proven effective in samples of the general public as a way to collect a majority of responses online while also providing an option for those unable to respond using the internet [63, 64]. There is some evidence that this approach may even be more effective than paper-only in certain populations who are accustomed to receiving similar communications from the surveyor via email [68]. Further, in a study of Dutch childhood cancer survivors, various strategies of offering a paper-based alternative to a web survey produced similar response rates [69]. Thus, using a paper follow-up response option to a web survey administered by a cancer registry may be an effective approach to obtain responses from those reluctant to respond to a web survey.

Overall, we did not find evidence that sending a brochure describing the cancer registry encouraged significantly more people to respond. However, the results suggest it could have varying effects across survey modes; while response to the paper survey was unchanged with its introduction, response was slightly higher (but not significantly so) for the web survey, thereby reducing the gap in response between the two modes. This also could be related to establishing legitimacy and trust, which may be especially helpful when asking people to respond online. Responding online entailed manually typing an unknown web address and entering an access code, and could possibly have been viewed with more suspicion or hesitation. There has been mixed evidence regarding the effect of sending study-specific brochures on study participation. Some studies have found no significant effect of brochures on response rates [70–72]. A recent analysis of multiple studies recruiting via the Utah Cancer Registry found that inclusion of a brochure describing details of the study in the recruitment packet decreased study cooperation [32]. However, study-specific brochures are very different in nature than the type used in this study, so it is unclear whether these past results are informative for cancer registry-specific informative brochure use. Due to the relatively small effect size of the brochure in this study, it would be worthwhile to retest this comparison in a larger sample size to further evaluate its effect across modes. It is worth noting that many registries require enclosure of such brochures in research recruitment mailings in order to explain how a person’s name was obtained. While our registry does not require a brochure, for registries that do, future testing may be made more informative by concentrating on variations in brochure design and contents rather than whether it is included or not.

In the comparison of individual demographics of respondents to nonrespondents, for the overall sample we found differences by time since diagnosis, age, and ethnicity. These differences are consistent with prior studies which have documented demographic factors that have influenced response to surveys or other studies administered via cancer registries include Hispanic ethnicity [73, 74], age [6, 7, 11, 21, 22, 32, 73, 75, 76], and time between diagnosis and recruitment [6, 11, 73, 77, 78]. Unlike prior studies, we did not observe overall differences according to sex [73, 74, 77], race [6, 7, 32, 73–75], or cancer stage at diagnosis [6, 7, 73]. However, we did see females overrepresented among respondents to paper but not web, and Hispanics and nonwhites underrepresented among web respondents. While we were unable to fully assess response by educational attainment, we did find that web and paper respondents reported similar levels of education.

There are limitations worth noting for this study. First, we were unable to offer incentives for participation, which resulted in a lower response rate than similar studies that have been conducted out of the registry. We were also unable to assess whether educational attainment or other socioeconomic variables affected response across the study arms because registries do not collect this information. Additionally, because this was a pilot study, the sample size was relatively small, making it difficult to identify significant differences for small effect sizes or to draw conclusions about various demographic subgroups included in the study. We also did not evaluate the use of a paper response option delivered later in administration, as is done in most web-push designs.

Another limitation is that due to the focus of this study on early-onset diagnoses of particular cancer sites, and the oversampling of some subgroups, our sample is not representative of cancer survivors generally. Most notably, in this study of early-onset cancer survivors, only 16.8% of the eligible sample was aged 60 or older; in contrast, 61.3% of all cancers of any site diagnosed in the same time period in Utah were among individuals aged 60 or above. Additionally, with the inclusion of two female-specific cancer sites and an oversample of Hispanics, compared to the entire registry of cancer diagnoses from 2001 to 2016, our sample had a higher percentage of females (56.5% vs. 48.4%) and Hispanics (15.5% vs. 5.8%) than are included in the database. Therefore, the results may not be generalizable to other registry-based study samples. In prior research, we found that recruitment outcomes from samples obtained from this registry vary according to cancer and demographic variables [32], thus we expect response results would vary in studies of different cancer sites or age groups. Nevertheless, the differences observed across experimental arms and demographic subgroups offer informative evidence and warrant further experimentation with web surveying in other registry samples.

## Conclusions

This study has found that collecting survey data via the internet is a feasible approach for cancer registries wishing to obtain responses from a representative group of individuals diagnosed with cancer, despite being limited to a postal mail and telephone-based contact approach. Although it may be advisable to utilize a paper follow-up response option for those who do not respond online (as is done in the standard web-push design), these results signal that registries may consider incorporating web surveys without much loss of overall response compared to the traditional paper-based approach. Future research should more fully assess the viability of a web-push design for obtaining survey data via cancer registries using larger samples that are inclusive of more cancer sites and older individuals. Additionally, further examination of how brochure enclosures influence response across survey modes is warranted.

## Supplementary information

**Additional file 1.** Study Questionnaire.

## Data Availability

The data analyzed for this study are not publicly available due to confidentiality considerations, but may be obtained upon request in accordance with Utah Cancer Registry Policies and Procedures for Data Disclosure. The study questionnaire is provided as a supplementary file, and recruitment materials are available from the corresponding author upon request.

## Abbreviations

Adj: Adjusted
CI: Confidence Interval
Col: Column
Ref: Referent
RR: Risk Ratio
SEER: Surveillance, Epidemiology, and End Results
