# Supplementary material for: The feasibility of web surveys for obtaining patient-reported outcomes from cancer survivors: a randomized experiment comparing survey modes and brochure enclosures
Source: BMC Med Res Methodol. 2019 Nov 15;19:208. doi: 10.1186/s12874-019-0859-9 (PMC6858678; doi:10.1186/s12874-019-0859-9)
Supplement: Supplementary file 1 — Additional file 1. Study Questionnaire. [file 12874_2019_859_MOESM1_ESM.pdf]

Assessing Cancer Survivors' Willingness to Participate in Research Studies

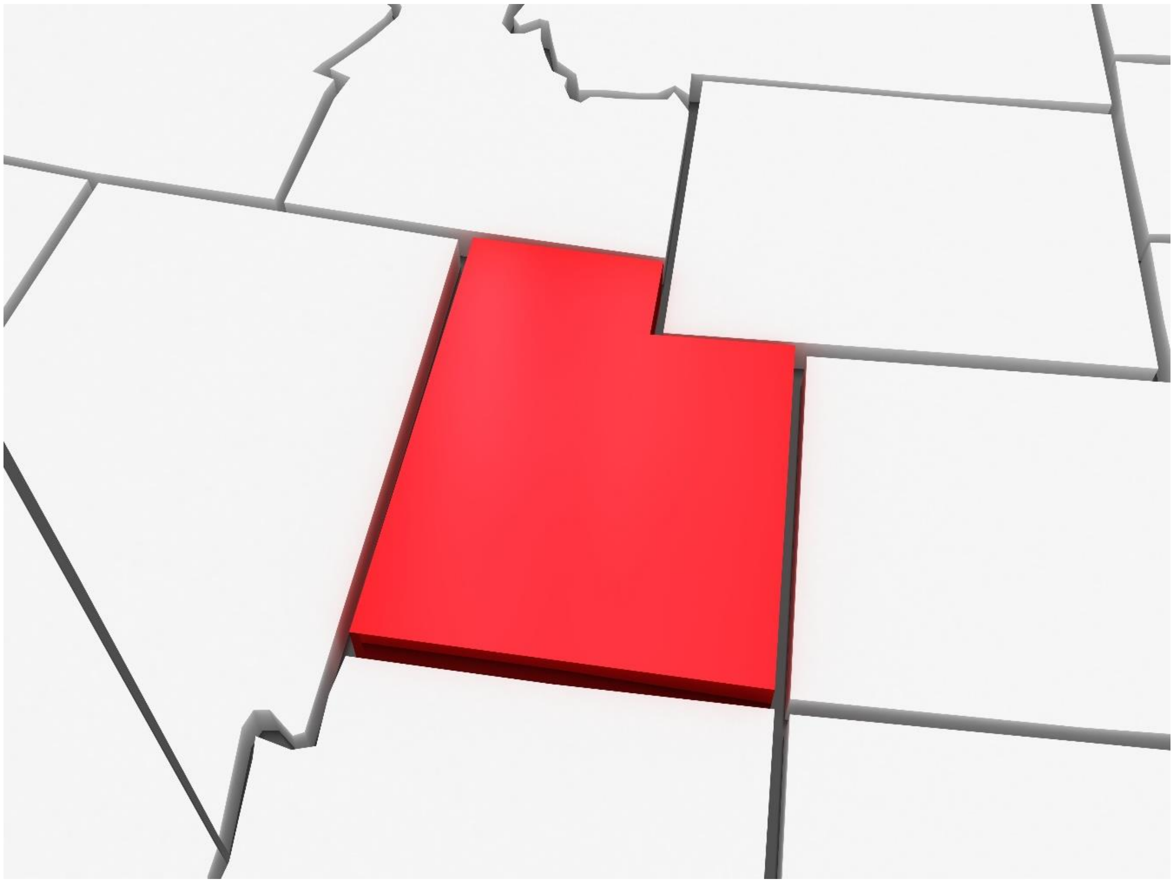

A study conducted by the Utah Cancer Registry and the National Cancer Institute

UNIVERSITY OF UTAH
HEALTH SCIENCES

Assessing Willingness to Participate in Cancer Research

Research Consent Information

The purpose of this study is to determine whether individuals who have been diagnosed with cancer may be willing to participate in a variety of types of cancer research studies in the future. We are seeking to understand the reasons why some people may participate in cancer research if they are asked, and why others may not. We are doing this study to better understand what types of studies could feasibly be done in the future.

We are asking you to complete a brief survey about your willingness to participate in cancer research. The survey has been developed by The National Cancer Institute (NCI), and is being conducted by the Utah Cancer Registry, located at the University of Utah. Other registries, including those in Detroit, Los Angeles, New Jersey, Iowa, and Louisiana, are also conducting this survey in their regions. Completing this questionnaire does not necessarily mean you will be asked to participate in other cancer research studies in the future, and **taking part in this survey does not mean you are agreeing to participate in future studies.**

The risks of this study are minimal. While we will make every effort to protect the confidentiality of your information, it is possible that a breach of confidentiality could occur. However, we will treat any information you choose to share with us as strictly confidential. All study documents will be kept in locked filing cabinets in a secured office, and shredded when the study is complete. Electronic records will be encrypted and stored on secured devices and servers in accordance with the State of Utah data security requirements. Only approved Utah Cancer Registry study staff will have access to complete study data. At the completion of the study, the Utah Cancer Registry will share the results and limited data with the National Cancer Institute, however, these data will **not** contain your name or any personally identifiable information about you. Any reports or publications resulting from this study will only contain summaries of results, not any information about individual participants.

If you have any questions about the study, or if you have complaints or feel you have been harmed by this research, please feel free to contact the study investigator, Dr. Morgan Millar from the Utah Cancer Registry, at morgan.millar@hsc.utah.edu or 801-587-0242.

You are encouraged to contact the University of Utah Institutional Review Board (IRB) if you have questions regarding your rights as a research participant. Also, contact the IRB if you have questions, complaints, or concerns which you do not feel you can discuss with the investigator. The University of Utah IRB may be reached by phone at 801-581-3655 or by email at irb@hsc.utah.edu.

It should take about 15 minutes to complete the questionnaire. Participation in this study is voluntary. You can choose not to take part. You can choose not to finish the questionnaire or skip any question you prefer not to answer without penalty or loss of benefits.

By completing and returning this questionnaire, you are giving your consent to participate. We thank you very much for considering our request.

University of Utah
Institutional Review Board
Exemption 12/27/2016

IRB_00096345

Survey to Assess Cancer Survivors' Willingness to Participate in Research Studies

Please enter today's date.

__ __ / __ __ / 2 0 __ __

m m / d d / y y y y

Questions About Your General Health

Q1. In general, would you say your health is: *Select one*

- ☐ Poor
- ☐ Fair
- ☐ Good
- ☐ Very good
- ☐ Excellent

Q2. Do you have any health problem for which you need to use special equipment, such as a cane, a wheelchair, a special bed, or a special phone? *Select one*

- ☐ Yes
- ☐ No

Q3. Have you ever been told by a doctor or other health professional that you have any of the following conditions? *If you are unsure or don't recognize the condition listed, please select "No"*

	Yes	No
A. Asthma, emphysema, or chronic bronchitis	☐	☐
B. Arthritis or rheumatism	☐	☐
C. A new cancer diagnosis since your original diagnosis	☐	☐
D. A recurrence of your cancer	☐	☐
E. Diabetes	☐	☐
F. Digestive problems (such as ulcer, colitis, or gallbladder disease)	☐	☐
G. Heart trouble (such as angina, heart attack, congestive heart failure, or coronary artery disease)	☐	☐
H. HIV illness or AIDS	☐	☐
I. Kidney disease	☐	☐
J. Liver problems (such as cirrhosis)	☐	☐
K. Stroke	☐	☐

Q4. Since your cancer diagnosis, how many different doctors or healthcare professionals (such as nurse practitioners) do you see on a <u>regular</u> basis? Please include providers you see for both cancer and non-cancer care. *Select one*

- ☐ 1
- ☐ 2 or 3
- ☐ 4 or 5
- ☐ 6 to 9
- ☐ 10 or more

Taking Part in Health Research

The next section asks questions about how likely you are to participate in <u>cancer research</u> and the type of information you might be willing to share when taking part in a study.

Q5. In the past, have you ever taken part in any kind of health research study, such as a clinical trial, survey, or study asking for a blood sample or other examinations? *Select one*

- ☐ Yes
- ☐ No ***IF NO, <u>SKIP TO QUESTION 6</u>***

Q5a. If yes, on a scale of 1-5, how was your experience in <u>the most recent study</u> in which you took part? *Select one*

- ☐ 1 Poor
- ☐ 2
- ☐ 3
- ☐ 4
- ☐ 5 Outstanding

Q5b. What did you do in <u>the most recent study</u> in which you took part? *Select <u>all</u> that apply*

- ☐ Completed interview(s) in person or over the phone
- ☐ Answered questionnaire(s) (either on paper or on a computer)
- ☐ Provided a blood, saliva, stool, or urine sample
- ☐ Had a visit at a clinic with some type of examination
- ☐ Took part in a clinical trial where you took medicine or received other treatments
- ☐ Other, *please specify:* ____________________

Q5c. In <u>the most recent study</u> in which you took part, did you receive compensation (for example, cash, a check, or gift cards) in appreciation of your time and effort? *Select one*

- ☐ Yes
- ☐ No

Q6. Many research studies require participants to fill out a survey with information on their medical history, their quality of life, and other factors. As part of a research study, would you be willing to fill out: *Select one*

- ☐ <u>Only one survey</u> sent to you at a single point in time (for example, 6 months after a cancer diagnosis)?
- ☐ <u>Multiple surveys</u> sent to you over an extended period of time (for example, once a year for 5 years)?
- ☐ I would not be willing to complete any type of survey ***<u>IF SO, SKIP TO QUESTION 7 ON NEXT PAGE</u>***

Q6a. How would you prefer to complete the survey(s)? *Select one*

- ☐ Interview by phone
- ☐ Paper-based by mail
- ☐ On a computer
- ☐ Smart phone or tablet (For example, an iPad)
- ☐ Other, *please specify:* ____________________

Q6b. If asked to complete an online/web questionnaire, which type of device do you think you would be most likely to use to complete it? *Select one*

- ☐ Home computer (desktop or laptop)
- ☐ Work computer (desktop or laptop)
- ☐ On a mobile device (cell phone/smart phone/tablet)
- ☐ Computer in a public place (library, community center, other)
- ☐ I would not complete an online questionnaire using any device

Q6c. What is the longest amount of time you would be willing to spend to complete a survey? *Select one*

- ☐ Less than 20 minutes
- ☐ 20 to 45 minutes
- ☐ 46 minutes to 1 hour
- ☐ More than one hour

Q6d. What language would you prefer for a written survey? *Select one*

- ☐ English
- ☐ Spanish
- ☐ Other, *please specify:* ____________

Q6e. What language do you prefer speaking on the phone? *Select one*

- ☐ English
- ☐ Spanish
- ☐ Other, *please specify:* ____________

Q7. Many research studies look at a patient's medical chart for information on cancer treatments, other health conditions, and medications. As part of a research study, would you be willing to allow qualified researchers to access your medical records, which would be kept confidential? *Select one*

- ☐ Yes
- ☐ No

Some research studies ask you to go to a specific location, such as a clinic or hospital, for an examination, interview, or tests. The next few questions ask about your ability and willingness to take part in a study in which you would be required to go to a clinic site near your home.

Q8. Would you take part in a study that requires a visit at a clinic site separate from a regular doctor's visit? *Select one*

- ☐ Yes
- ☐ No ***IF NO, <u>SKIP TO QUESTION 9 ON NEXT PAGE</u>***

Q8a. Would you need help with transportation to get there? *Select one*

- ☐ Yes
- ☐ No

Q8b. What is the maximum traveling time that you would be willing to spend to get to a clinic site for a research study? *Select one*

- ☐ Less than 10 minutes
- ☐ 10 to 29 minutes
- ☐ 30 to 59 minutes
- ☐ 1 hour or more

Q9. Would you be willing to take part in a study if the examination was part of your regular doctor's visit (and did not require a special trip)? *Select one*

- ☐ Yes
- ☐ No

Q10. What is your usual mode of transportation to doctors' appointments? *Select one*

- ☐ I drive a car or other type of vehicle
- ☐ A friend or family member drives me
- ☐ Public transportation
- ☐ Other, *please specify:* __________

Q11. Cancer research studies may ask information about your health, concerns, support, and other survivorship experiences to help researchers better understand the challenges and behaviors that people with cancer face, or how they respond to treatment.

As a part of a research study, would you be willing to answer questions about your:

	Yes	No
A. Symptoms experienced during and after cancer treatment?	☐	☐
B. General quality of life (such as your feelings of well-being and health)?	☐	☐
C. Lifestyle (such as what you eat, how much alcohol you drink, if you smoke cigarettes, or how often you exercise)?	☐	☐
D. Social network (such as whether you have people to support you when you feel down)?	☐	☐
E. Finances (such as whether you have large debts from treatment costs)?	☐	☐
F. Interactions with healthcare providers such as doctors and nurses?	☐	☐
G. Are there other issues that you think would be important to study? *If yes, please specify below:* __________	☐	☐

Q12. Some cancer research studies look at substances in the blood or other bodily samples that may be related to cancer or effects of treatment. Although we are not asking you to provide samples now, the next questions ask about your willingness to provide different types of samples (at no cost to you) as part of a research study.

As a part of a research study, would you be willing to:

	Yes	No
A. Provide a <u>blood sample</u>, collected either at a clinic visit or at your home during a visit by a health professional?	▢	▢
B. Provide a <u>saliva sample</u>, collected either at a clinic visit or at your home using a kit and mailed to investigators?	▢	▢
C. Provide a <u>urine sample</u>, collected either at a clinic visit or at your home using a kit and mailed to investigators?	▢	▢
D. Provide a <u>stool sample</u>, collected either at a clinic visit or at your home using a kit and mailed to investigators?	▢	▢
E. Would you give permission for researchers to access some of the tissue taken at the time of your surgery (hospitals routinely preserve and keep some of the tissue removed at surgery)?<br>▢ I did not have surgery related to cancer	▢	▢

Q13. Samples that are collected as part of a study may be used to examine genetic material (your DNA) that may give information about the cancer you had or the effects of treatment. This information could be shared confidentially with qualified researchers. Would you be willing to allow cancer researchers to study your DNA or genetic material from samples? *Select one*

- ▢ Yes
- ▢ No

Q14. People have various reasons why they may take part in a study. What are the main reasons you would be interested in participating in a cancer research study? *Select <u>all</u> that apply*

- ▢ I want to give back to the medical community and help those with cancer in the future
- ▢ I would like to learn more about cancer and relevant resources such as clinical trials
- ▢ I am interested in available compensation
- ▢ Other, *please specify:* ____________
- ▢ None, I would not be interested in participating in a cancer research study for any reason

Q15. Which of the following might prevent you from taking part in a cancer research study? *Select <u>all</u> that apply*

- ▢ Takes too much time or effort
- ▢ Concern about my privacy/confidentiality of information
- ▢ I am not interested in research
- ▢ I do not know or trust researchers
- ▢ It would be too emotionally difficult for me to think about my cancer experience at this time
- ▢ Other, *please specify:* ____________
- ▢ None of these would prevent me from taking part in a cancer research study

Q16. Would any of the following make you more likely to participate in a cancer research study? *Select all that apply*

- ☐ Travel assistance/parking validation
- ☐ Getting back your study results
- ☐ Gift cards or other compensation
- ☐ If your doctor endorsed the research study
- ☐ Being assured that your information will remain private
- ☐ A newsletter with updates about general health information found in the study
- ☐ Knowledge that your contribution could benefit others with cancer in the future
- ☐ Other, *please specify:* __________
- ☐ None of the above

Family Questions

Q17. When people with health problems need help or care, sometimes a family member or friend provides assistance. Who has helped you, or would most likely help or take care of you, if you needed it? *Select all that apply*

- ☐ Spouse/partner
- ☐ A daughter or son
- ☐ Another family member
- ☐ A friend or neighbor
- ☐ Other, *please specify:* __________
- ☐ I don't know who I could ask to help me

Q18. Would you be willing to ask a family member or friend to take part in a study with you? *Select one*

- ☐ Yes
- ☐ No

Q19. Do you think this family member or friend would agree to take part in a study? *Select one*

- ☐ Yes
- ☐ No

Tell Us About You

Finally, we'd like to ask just a few more questions to make sure we are hearing from a variety of different people.

Q20. What is the highest level of education you have completed? *Select one*

- ☐ Grade school or less
- ☐ Some high school or technical school, no degree
- ☐ High school graduate or GED, includes technical school graduate
- ☐ Some college, no degree
- ☐ College graduate, four-year degree
- ☐ Graduate or professional degree
- ☐ Other, *please specify:* __________

Q21. What is your ethnicity? *Select the <u>one</u> that best describes you*

- ☐ Hispanic
- ☐ Non-Hispanic

Q22. What is your race? *Select <u>all</u> that describe you*

- ☐ White/Caucasian
- ☐ Black/African-American
- ☐ Asian, *please specify country or countries of origin:* ______
- ☐ American Indian or Alaska Native
- ☐ Native Hawaiian or other Pacific Islander, *please specify island(s) of origin below:* ______
- ☐ Other, *please specify:* ______

Q23. Are you <u>presently</u>: *Select <u>all</u> that describe you*

- ☐ Employed full-time (working 35-40 hours per week)
- ☐ Employed part-time (working less than 35 hours per week)
- ☐ A full-time homemaker
- ☐ Full or part-time student
- ☐ Retired
- ☐ Disabled or on medical leave
- ☐ Unemployed
- ☐ Looking for work

Q24. What kind of health insurance or health care coverage do you have now? *Select <u>all</u> that apply*

- ☐ HMO plan with specific clinics and hospitals
- ☐ Other private insurance (such as Blue Cross/Blue Shield, Aetna, etc.)
- ☐ Medicare
- ☐ Medigap
- ☐ Medicaid or other state-sponsored plan
- ☐ Military Health Care (TRICARE, VA, CHAMP-VA)
- ☐ Indian Health Service or Tribal Health Service
- ☐ Other, *please specify:* ______
- ☐ No coverage of any type

Q25. Since your cancer diagnosis, have you experienced any periods without health insurance or health care coverage? *Select one*

- ☐ Yes
- ☐ No

Thank you for taking the time to answer these questions today.

On the next page, you'll find space to share any other comments you may have.

If you have any other thoughts or comments about taking part in research that you would like us to know, please share them here.

Questions?

If you have any questions, you may contact us by phone at
801-585-3317 or by email at ucr_research@hsc.utah.edu.

Please return your completed questionnaire to us using the stamped envelope provided.

If the envelope has been misplaced, please send your completed questionnaire to:

Utah Cancer Registry
650 Komas Drive, Suite 106B
Salt Lake City, UT 84108

UNIVERSITY OF UTAH
HEALTH SCIENCES
